# Exploring Predictors of Preclinical Performance in Predoctoral Operative Simulation Courses

**DOI:** 10.1002/jdd.70022

**Published:** 2025-09-01

**Authors:** Nicholas DePinto, Emily H. Sabato, Donald P. Lapine, Eileen R. Hoskin, Herminio L. Perez, Shuying S. Jiang, Rosa Chaviano‐Moran

**Affiliations:** ^1^ Department of Restorative Dentistry Rutgers School of Dental Medicine Newark New Jersey USA; ^2^ Rutgers School of Dental Medicine Newark New Jersey USA; ^3^ Department of Academic Affairs Rutgers School of Dental Medicine Newark New Jersey USA

**Keywords:** dental school admissions, dental admission test, operative dentistry, perceptual ability test, predoctoral education

## 1 Introduction

Dental school presents a unique challenge as students must not only master rigorous academic didactic requirements but also the essential hand skills critical to clinical dental practice. This dual demand poses a significant challenge for both admission committees striving to evaluate applicants’ academic and manual potential and for faculty tasked with teaching foundational clinical skills early in the predoctoral curriculum.

Admissions processes aim to identify candidates using standardized tests and hands‐on evaluations [1]. Admissions committees often find it challenging to identify applicants with robust critical thinking skills and the potential for excellent clinical skills [2]. Many studies have sought to identify predictors of student performance in dental school. The Dental Admission Test (DAT) is a standardized exam used for admissions to dental school in the United States and Canada. It assesses a candidate's scientific knowledge, perceptual skills, and overall academic ability to succeed in dental school. Overall, DAT scores have been found to modestly predict positive performance across the first year of the dental curriculum [3, 4].

Several sections of the DAT have been found to predict performance in dental school. The Perceptual Ability Test (PAT) measures spatial visualization and manipulation skills, which are crucial for cavity preparations and other clinical dental procedures. Performance on the PAT was positively correlated with performance in the first semester of dental school [5]. In addition to echoing the positive correlation of the PAT to success in dental school, research at a different US dental school showed Reading Comprehension (RC) to correlate to positive outcomes in preclinical and clinical coursework [6]. The Quantitative Reasoning (QR) section assesses fundamental mathematical skills and statistics. Strong performance on the QR section may indicate a candidate's ability to think critically, handle complex data, and apply that knowledge. Performance in this section could serve as a predictive indicator of a student's ability to become an analytic thinker [6], a skill essential for applying didactic knowledge in laboratory and clinical settings.

Research has also explored the relationship between admissions criteria and preclinical coursework. Some research suggests that dexterity tests do not reliably predict dental school performance [7]. However, a 2015 study found a positive relationship between scores in chalk carving exercises and performance in preclinical operative laboratory courses [8]. Further, a study found performance on wax carving exercises and high PAT scores to be positively associated with performance in introductory restorative dentistry preclinical laboratory courses [9]. A positive relationship between PAT scores and student performance in early preclinical prosthodontics laboratory coursework has been found [10]. This relationship between PAT scores and preclinical outcomes is also observed in an evaluation of outcomes of examinations on Classes II and III restorations and preparations [1]. Studies have also sought to determine the specific PAT score thresholds that may predict success in clinical coursework; minimum PAT scores of 15 [11] and 23 [9] are predictive in different studies.

Rutgers School of Dental Medicine (RSDM) has also previously conducted investigations into predictors of student performance in the preclinical courses. For example, a 2018 study compared students' performance in operative preclinical laboratory courses to their DAT results. This investigation found that a one‐unit decrease in PAT was related to a 43% increase in the odds of failing the first preclinical lab operative course and similar negative relationships in other preclinical lab courses [12]. However, other research at RSDM has found that other admission factors, such as GPA and noncognitive skills, may contribute significantly to predicting student success [13].

The RSDM preclinical curriculum has been significantly revised since the aforementioned study of preclinical outcomes, with the introduction of mandatory in‐course remediation of failed practical examinations as well as changes to the grading rubrics. This investigation builds upon prior research [12] and seeks to add to the existing body of knowledge regarding student performance in introductory preclinical simulation courses. This manuscript details the revised preclinical operative curriculum at RSDM , analyzes student outcomes in these courses compared to their admission scores, and discusses potential avenues for optimizing student success. A primary aim is to understand the relationship between dental students' DAT scores, their undergraduate Grade Point Average (GPA) and undergraduate science GPA, and their performance on dental school's preclinical operative dentistry practical exam scores under the current preclinical operative curriculum. As this analysis utilizes numeric practical score grades rather than letter grades, the analysis offers a level of detail unavailable in the study at our institution in 2018. This information is intended to enhance the body of knowledge supporting the decisions of dental admission officers, student support personnel, and course directors to promote positive student outcomes in dental school.

### 1.1 Preclinical Operative Curricula at RSDM

At RSDM, the operative preclinical curriculum begins in the first term and continues through the second year, culminating in the students' first patient care experiences. These hands‐on laboratory courses are time intensive, accounting for 30% of the preclinical laboratory curriculum and developing foundational hand skills for clinical practice.

Over the D1 and D2 years, RSDM predoctoral students take three operative simulation (preclinical) laboratory courses, which were restructured in the 2019–2020 academic year. Preclinical Operative Dentistry I and II (PODI and PODII) are administered during the D1 year, and Preclinical Operative Dentistry III (PODIII) is administered during the D2 year. A standardized series of rubrics is utilized across these courses during projects for both student self‐assessment and faculty feedback, and used for grading of practical examinations. In‐course remediation is required for failed practicals.

PODI begins with basic cavity preparation design and restorations, covering Classes I, III, and V. PODII focuses on the more challenging Class II preparations and restorations. This classification typically requires repetition to master and is a requirement for licensing exams. PODIII covers Class II, III, IV, and V preparations and restorations. The complex indirect restoration, an onlay, is also addressed in PODIII. As the curriculum advances, students are expected to master skills in a shorter time frame. The rubrics in PODIII also introduce the critical error; as the critical error is an automatic failure, this raises the expectations of the assessment for students approaching entry to the patient care clinic. Together, these three preclinical operative dentistry courses teach and evaluate students on diverse and complex skills prior to their engagement in patient care as the operator.

## 2 Methods

This study was approved by the RSDM IRB(PRO 2024000621). This study employs a retrospective cohort design, reviewing all students enrolled in the traditional four‐year Doctor of Dental Medicine (DMD) program at RSDM in the matriculating classes of 2023, 2024, 2025, and 2026. The students' admission records, including subscores for the DAT and undergraduate cumulative and science GPAs were collected from the student record system. Numerical scores for each practical examination were retrieved from the learning management system. Numeric scores for failed practical examinations were not maintained in the learning management system and thus were unavailable for retrospective review. All students who failed on the first attempt of a practical exam passed on the second attempt; a maximum score of 70 was recorded for the second attempt and utilized for this analysis.

### 2.1 Study Population

The initial study population was 362. Students who did not complete the first 2 years of the curriculum with the same class with which they began were excluded from the analysis. Five students took a leave of absence and returned to a later class, five entered the 5‐year predoctoral program, two repeated the first or second year of the curriculum, five withdrew, and 10 were dismissed from RSDM, resulting in a final study population of 335. It should be noted that students excluded from this analysis due to their inability to progress with their class had no difficulty with operative preclinical laboratory coursework based on their recorded grades. Of this population, one student was withdrawn in the fourth year, and one took a leave of absence in the third year; these students were retained in the study as these events were outside of the academic period of interest.

### 2.2 Statistical Methods

Predictor variables included undergraduate Science GPA and undergraduate cumulative GPA (UG GPA) and the elements of the DAT scores: Academic Average (AA), Perceptive Aptitude Test (PAT), QR, RC, Biology (BI), Inorganic Chemistry (IC), Organic Chemistry (OC), and Total Science (TS). Averages of practical examinations in the preclinical laboratory courses were utilized to develop the five outcomes variables: PODI average, PODII average, PODIII average, D1 overall (including PODI and PODII practical exams), and D1 and D2 overall (including practical exams for all three courses). Due to the curriculum structure, the PODIII average represents the entirety of the preclinical operative simulation coursework in the second year (D2 overall average).

Descriptive statistics were compiled, and bivariate analyses were conducted to explore whether each predictor variable was related to each outcome. The Pearson correlation coefficient was used to assess the correlation between the two interval variables. Finally, regression analyses were performed for each outcome. The predictor variables with a *p* value less than 0.10 in the bivariate analysis were included in the regression model. Linear regression models were used for all outcomes. SPSS Statistic 29 software (IBM Corp., Armonk, NY, USA) was used for the data analysis. The statistical significance level (two‐sided) was set at *p* < 0.05 for all tests. In addition, student performance in Operative I, II, and III preclinical simulation laboratory was compared.

## 3 Results

Descriptive statistics are detailed in Table 1. Bivariate correlations are detailed in Table 2. PAT was significantly correlated with all five outcomes (PODI average, PODII average, D1 overall, PODIII average, and D1 and D2 overall) when the bivariate analyses were performed and remained significant in the regression models for all five outcomes. QR was significantly correlated with PODI average and D1 overall outcomes when the bivariate analyses were performed but was no longer significant in the regression model.

**TABLE 1:** GPA and DAT scores and average scores across summative (“practical”) assessments.

Class	*N*	Sci GPA	Cum UG GPA	AA	PAT	QR	RC	BI	IC	OC	TS	Operative I	Operative II	D1 overall	Operative III/D2 overall	D1 and D2 overall
2023	80	3.53	3.60	21.9	20.8	20.2	21.4	22.1	22.6	23.3	22.4	83.55	90.01	86.14	90.97	88.55
2024	88	3.56	3.62	21.5	20.3	20.4	22.0	21.1	22.1	22.2	21.4	85.23	81.34	83.34	88.00	85.43
2025	85	3.52	3.58	21.9	21.4	21.0	22.0	21.4	22.6	22.5	21.9	83.31	83.99	83.70	88.16	85.56
2026	82	3.60	3.64	21.3	21.4	21.6	22.1	20.4	20.2	21.3	21.5	83.56	85.97	84.93	88.40	86.38
Overall	335	3.55	3.61	21.7	21.0	20.8	21.9	21.3	21.9	22.3	21.8	83.93	85.21	84.47	88.85	86.44
Range	—	2.19–4.00	2.56–4.00	17–28	14–30	9–30	13–30	15–30	14–30	15–30	16–30	70–94.8	70–98.5	70–94.8	70–97.6	70–95.5

**TABLE 2:** Bivariate correlations.

	Operative I average	Operative II average	D1 Overall	Operative III average and D2	D1 and D2 overall
	Pearson correlation coefficient (*p* value)				
Academic average	0.057 (0.301)	0.040 (0.463)	0.051 (0.348)	0.033 (0.544)	0.052 (0.347)
PAT average	0.168 **(0.002** ^*^ **)**	0.145 **(0.008** ^*^)	0.192 **(< 0.001** ^*^)	0.180 **(< 0.001** ^*^)	0.200 **(< 0.001** ^*^)
Quant reasoning	0.119 (**0.030** ^*^)	0.063 (0.249)	0.112 **(0.041** ^*^)	0.027 (0.618)	0.081 (0.142)
Reading comprehension	0.036 (0.510)	−0.026 (0.634)	0.010 (0.854)	−0.036 (0.506)	−0.013 (0.820)
Biology	0.050 (0.359)	0.062 (0.257)	0.054 (0.321)	0.051 (0.352)	0.065 (0.232)
Chem‐inorganic	−0.020 (0.714)	0.021 (0.706)	−0.007 (0.902)	0.012 (0.820)	0.007 (0.895)
Chem‐organic	−0.013 (0.813)	0.017 (0.760)	−0.012 (0.827)	0.090 (0.102)	0.041 (0.450)
Total science	0.018 (0.736)	0.081 (0.139)	0.052 (0.341)	0.044 (0.427)	0.059 (0.280)
Overall result	0.060 (0.277)	0.067 (0.225)	0.069 (0.208)	0.061 (0.264)	0.076 (0.167)
Sci GPA	−0.011 (0.838)	0.042 (0.449)	0.022 (0.692)	−0.010 (0.857)	0.013 (0.809)
CUM UG GPA	−0.008 (0.884)	0.080 (0.144)	0.049 (0.372)	0.015 (0.782)	0.042 (0.441)

* Statistical significance at *p* < .05.

The result of regression analysis is also shown in Table 3. A one‐unit score increase in the PAT was associated with a 0.27, 0.33, 0.30, 0.28, and 0.31 increase in the PODI average, PODII average, D1 overall, PODIII average, and D1 and D2 overall grades, respectively.

**TABLE 3:** Results of linear regressions.

Dependent variables	Predictor variables	Unstandardized Coefficients	Std. error	*p* value	*R*‐square
Operative I average	(Constant)	76.58	2.29	< 0.001	0.031
	PAT average	0.27	0.12	0.018	
	Quant reasoning	0.08	0.09	0.407	
Operative II average	(Constant)	78.35	2.59	< 0.001	0.021
	PAT average	0.33	0.12	0.008	
D1 overall	(Constant)	77.25	2.09	< 0.001	0.037
	PAT average	0.30	0.11	0.004	
	Quant reasoning	0.04	0.08	0.626	
Operative III average and D2	(Constant)	82.92	1.78	< 0.001	0.033
	PAT average	0.28	0.08	< 0.001	
D1 and D2 overall	(Constant)	80.05	1.73	< 0.001	0.040
	PAT average	0.31	0.08	< 0.001	

The results of comparing overall student averages in PODI and PODIII are detailed in Table 4. In addition, this table shows the percent change in student scores for each student from PODI to PODIII, with an analysis of the statistical significance of the change.

**TABLE 4:** Comparisons of results between operative preclinical courses.

Paired Sample *T*‐test	*N*	Mean	SD	*p* value
Operative I average	335	83.93	5.20	< 0.001^*^
Operative III average	335	88.85	4.34	

**Mean difference**	*N*	Mean	SD	95% CI	*p* value
Operative III − Operative I	335	4.91	4.66	(4.41, 5.41)	< 0.001^*^

* statistical significance at *p* < .05.

## 4 Discussion

This retrospective cohort study investigates the relationship between admission metrics and preclinical performance among students enrolled in RSDM's Doctor of Dental Medicine program across four cohorts (entering classes of 2023–2026). The study provides a robust view of potential predictive factors influencing performance in critical preclinical operative courses by examining 4 years of data for students who took these courses with the integrated remediation and self‐assessment protocols.

The analysis demonstrates a positive relationship between PAT score and performance in RSDM’s preclinical operative coursework. The increase is consistent in size and statistically significant across the outcome variables. This finding supports previous research suggesting a relationship between PAT scores and performance in preclinical laboratory coursework [1, 9, 10, 11]. However, it must be noted that the increase is a modest 0.31 points on a 100‐point grading scale for the practical assessment averages across the three courses (*p* < 0.001).

Analysis of the students' scores in PODI compared to PODIII demonstrates that the students perform significantly better in PODIII (Table 4). The average grades rise by a mean of 4.91 points from PODI in the D1 year to PODIII in the D2 year. This supports the importance of developing strategies that support the development of students' hand skills from the moment of matriculation, through the rigors of preclinical coursework and into clinical training, ultimately preparing them for future independent patient care.

### 4.1 Implications for Admissions Personnel

Our findings contribute to a growing body of evidence on admissions criteria in dental education, providing insights into which factors are most predictive of preclinical success. Earlier research at RSDM and other institutions demonstrated a relationship between several DAT subscores (e.g., PAT, AA, RC) to student performance in the first year and cumulatively across the curriculum [1, 3, 14]. This study underscores the importance of PAT scores as a moderate predictor of performance in preclinical operative courses, suggesting a significant, though limited, role in identifying spatial abilities applicable to practical dental skills [5, 9, 11, 12].

In addition, QR was also found to be statistically related to positive outcomes on comprehensive examinations in prior research [6]. Our analysis found QR significant in the bivariate correlation for outcomes in the PODI (*p* = 0.002) and for the average of the PODI and PODII courses (*p* = 0.041). It has also been found to be weakly associated with performance in a manual dexterity exercise at another institution [9]. Together, these results suggest QR may be a subscore of value in admissions decisions.

Although these subscores have modest predictive value, they are only one aspect of a comprehensive assessment process that must balance cognitive, spatial, and manual dexterity skills to identify students best suited for the demands of dentistry. The scope of dental education is broader than that of preclinical laboratory, and success cannot be universally predicted by qualitative variables [11, 15]. Accordingly, while they should be weighed, it is not advisable for admission committees to place overt emphasis on a particular subscore of the DAT. The modest correlation of PAT score to preclinical outcomes suggests that spatial ability is only one component of preclinical success and that other factors may play a substantial role [12]. Other cognitive variables, such as the development of self‐assessment ability and level of comfort with indirect vision, also play a role, particularly as students progress through increasingly complex tasks and transition to a clinical environment, with additional factors influencing outcomes [15].

### 4.2 Implications for Student Support

Prior research, including studies conducted at RSDM, indicates that high PAT scores often correlate with success in preclinical coursework, particularly in operative and prosthodontics laboratory work [5, 12]. A 2022 study comparing PAT scores to outcomes in an operative preclinical course at another US dental school had comparable results, suggesting awareness of this finding would be beneficial for focusing on students who may begin dental school at a deficit and creating programming for this [1].

Support in developing hand‐eye coordination for dentistry skills may benefit students with a lower inherent aptitude for preclinical laboratory work [9]. Awareness of this positive correlation of PAT scores to preclinical skills could trigger proactive availability of additional assistance in preclinical laboratory work for better outcomes. Previous studies have suggested a strong correlation between PAT scores and performance in early clinical tasks, with PAT thresholds of 15 and 23 identified as indicative of future success in dental school [9, 11]. While it is generally prudent to make extra support, such as tutoring, available to all students [16], these identified students could receive additional communication encouraging the utilization of services. For more intensive intervention, a course in developing hand skills pre‐matriculation could be created. In addition, other studies have suggested that one of the main barriers to preclinical learning for novice dental students is the use of indirect vision [17]. When students are first cultivating this skill, instructors perform chairside demonstrations for all students; ultimately, the development of an indirect vision skillset is achieved through repetition and practice. At RSDM, indirect vision skills are formally introduced early in the preclinical operative curriculum through dedicated lab exercises that isolate mirror use and emphasize spatial orientation. Faculty members provide chairside demonstrations and structured feedback during these exercises and demonstrate different ways of mirror use to support development of indirect vision skills among all students. An exercise at the beginning of preclinical coursework assessing hand skills with indirect vision would be most beneficial to identifying students who may initially struggle [17].

### 4.3 Summary of RSDM’s Preclinical Operative Laboratory Structure Supporting Development of Clinical Skills

RSDM's curriculum for preclinical operative dentistry focuses on structured grading, self‐assessment, and objective remediation. As previously noted, students excluded from the study due to failure to progress with their entering cohort had no documented challenges in preclinical laboratory courses, suggesting the study's retained sample represents a reasonable comparison of admission factors to preclinical outcomes. This suggests that the structure of RSDM’s preclinical curriculum may provide adequate support to all students in developing their skills relevant to operative dentistry. This section details this structure, which may serve as a recommended framework for other institutions. Students with admission factors that may indicate the need for extra support may significantly benefit from the implemented changes in RSDM’s operative preclinical curriculum.

#### 4.3.1 Formative Skill Development

Structure and requirements are consistent across the three preclinical operative dentistry courses, promoting formative development of students' hand skills. The courses use the same cavity preparation classification‐specific rubrics, with the introduction of the “critical error” in the PODIII course. This criterion is common to board exams and often aligns with what would result in an unsuccessful outcome if performed on a patient. These consistent grading rubrics represent a crucial approach to enhancing the objectivity of formative and summative assessments. The rubrics have been established with clearly defined criteria so that evaluating instructors can significantly reduce variations or inconsistencies in student feedback, making the evaluation process more predictable and reliable.

Each course includes a series of projects with defined completion deadlines. Students must self‐evaluate the projects utilizing the grading rubrics. Conducting a self‐assessment is an essential component of the course design and is completed prior to instructor evaluation. Repeated application of rubrics across all cavity classifications helps students to build and refine their reflective self‐assessment skills and deepens their understanding of where their skills may be lacking or need improvement. This self‐assessment process bridges the gap between student perception and evaluator expectations. When students have already self‐assessed, instructor feedback may be more impactful [18]. As students enhance their self‐assessment skills, they begin to correct errors prior to faculty evaluation; consistent use of self‐assessment utilizing course grading rubrics has been shown to improve student outcomes in preclinical simulation coursework [, 19].

Calibrated faculty evaluate the project and indicate when the work is satisfactory as per the rubric expectations. Students may repeat projects as many times as necessary to achieve a positive formative outcome without penalty. All projects must be successfully completed prior to attempting the practical examinations. Students who have not completed their assignments, including passing the faculty evaluation, prior to the practical examination earn a grade of zero and fail the course.

#### 4.3.2 Summative Evaluation and In‐Course Remediation

All three courses require passing on all summative evaluations, referred to as Practical Exams. All students who receive less than an 80 on any practical must review that exam with the course director to ensure a clear understanding of the errors and formulate a plan to help ensure better outcomes in the future. This allows for identification and intervention for any student who may be struggling. This is of particular value for those who perform poorly on the first practical exam. A student who fails a Practical Exam must complete in‐course remediation and retake the examination. This remediation includes meeting with the course director to review deficiencies and successful completion of three exercises reflecting the content of the practical; these exercises must be reviewed and approved by a faculty member assigned to the course. This remediation must be completed prior to repeating the examination. The highest score the student receives for this summative assessment is 70. If unsuccessful in the re‐take exam, the student will fail the course regardless of performance on other practical examinations.

### 4.4 Faculty Calibration

RSDM employs a robust faculty calibration program to ensure consistency in student learning and grading. The course directors for POD I, II, and III were consistent across the analysis period; each course was directed by a member of the Department of Restorative Dentistry. In addition, the school's Director of Operative Dentistry, who oversees the POD course series, was in this position throughout the analysis period. As the value of consistent feedback and grading is known, efforts are made during the faculty assignment process to maintain the consistency of faculty assigned to the POD courses across academic years [20].

Faculty calibration is conducted annually for all faculty. Calibration occurs by first having the faculty review the objective written and visual rubrics to familiarize themselves with the grading protocol. Next, faculty grade a series of projects that students have recently completed using the rubrics. These grading results are compared across faculty, and variations in grading are detected and discussed. The course director reviews the grading results to ensure that faculty grading is consistent. New faculty entering the course complete the same calibration grading exercise, working alongside experienced faculty for initial project assessments and practical exam grading. Once the new faculty member's grades align within five points of both the course director's and experienced faculty's assessments, they are approved to grade independently.

To help verify grading of practical exams reflects calibration, a multi‐step approach is utilized for grading of practical examinations. All practical grading is blinded to prevent unintentional bias. Each student's practical exam is graded by a single faculty member; each faculty grades examinations for a portion of the class. Practical examinations receiving an unsatisfactory score are graded by a second faculty member in the course. Then, the course director reviews both grade results and makes a final determination. In addition, the course director does a final review of all practical exam results to determine consistency and to check for any possible faculty variations in grading.

#### 4.4.1 Student Support Services for Preclinical Coursework

Students have access to the preclinical laboratory 24 h a day, 7 days a week—outside of scheduled class times—to facilitate practice as their schedule permits. RSDM offers twice‐weekly peer tutoring sessions for preclinical laboratory coursework. These sessions are offered at no cost to all first‐ and second‐year students; the school pays the tutors. Students with deficiencies in their preclinical coursework are strongly recommended to attend. These sessions are staffed by fourth‐year students selected for their clinical excellence. Course directors calibrate tutors on the grading rubrics, and updates are provided to the faculty regarding the progress and struggles of the students attending tutoring. This practice assists course directors and faculty in providing specific attention and support to students with deficiencies.

### 4.5 Limitations of the Study and Suggestions for Future Research

An inherent limitation of this study is the relatively small sample size, capturing four cohorts at one US dental school. The lack of numerical data regarding those who failed the practical on the first attempt is an additional limitation of this analysis. Course policy mandates a retake for a student failing a practical, and all students who failed a practical earned the maximum score of 70 on any retake they attempted in the study period, which is the grade recorded in the course records. In addition to the simple fact that the practical failure data was not retained for these students, students who had a critical error automatically failed the practical; no numeric score was calculated or recorded for these failures. If this study were replicated in the future, it would be worthwhile to intentionally record data of this nature and conduct a similar analysis utilizing numeric failure grades on the first attempt of a summative practical exam only. An analysis of this nature may highlight the impact of the mandatory in‐course remediation protocols for students with lower PAT scores or other noteworthy quantitative admissions factors. The curriculum revisions may improve student outcomes independent of their admission factors. Notwithstanding, the data analysis retains merit, as the limitation of a score of 70 on a repeat exam does reflect a marginal performance. Furthermore, the numeric scoring protocol lends itself well to statistical analysis.

Future research should also consider an analysis of the efficacy of student support in preclinical laboratory coursework, such as the impact of the utilization of peer tutoring as compared to student admission scores and outcomes in preclinical coursework. Additional research comparing PAT scores to numeric practical assessment scores across the range of preclinical coursework, including dental anatomy and prosthodontics courses, would also be beneficial, both in evaluating the value of the PAT in admissions and for the development of support services for preclinical laboratory education. Studies that examine whether students who enter the class with lower aforementioned admission indicators and less dexterity may eventually, with experience, attain the same level of preclinical operative preparation skills due to the curriculum updates and support services would be a worthy endeavor.

Furthermore, given the limited value of standardized assessments in predicting student outcomes in preclinical operative dentistry, future research exploring the relationship between noncognitive metrics such as resilience may be warranted to inform holistic admissions processes [12]. In addition, a comprehensive evaluation of preclinical laboratory outcomes utilizing the new scoring scale for the DAT, which will have a significantly more detailed range, is likely to have significant value.

## 5 Conclusion

The research highlights the modest predictive value of the PAT for success in operative preclinical laboratory coursework. These results underscore the importance of qualitative metrics as a component of individualized admissions assessments. Given the outcomes of this curriculum, the development of integrated support services for the development of clinical hand skills is recommended. Identification of individuals in need of early intervention and support services is a potential benefit.
